# Hepatic Steatosis Is Associated with High White Blood Cell and Platelet Counts

**DOI:** 10.3390/biomedicines10040892

**Published:** 2022-04-13

**Authors:** Yu-Lin Chao, Pei-Yu Wu, Jiun-Chi Huang, Yi-Wen Chiu, Jia-Jung Lee, Szu-Chia Chen, Jer-Ming Chang, Shang-Jyh Hwang, Hung-Chun Chen

**Affiliations:** 1 Division of Nephrology, Department of Internal Medicine, Kaohsiung Medical University Hospital, Kaohsiung Medical University, Kaohsiung 807, Taiwan; leonchuo@gmail.com (Y.-L.C.); wpuw17@gmail.com (P.-Y.W.); karajan77@gmail.com (J.-C.H.); chiuyiwen@kmu.edu.tw (Y.-W.C.); u9051108@kmu.edu.tw (J.-J.L.); sjhwang@kmu.edu.tw (S.-J.H.); chenhc@kmu.edu.tw (H.-C.C.); 2 Department of Internal Medicine, Kaohsiung Municipal Siaogang Hospital, Kaohsiung Medical University, Kaohsiung 812, Taiwan; 3 Faculty of Medicine, College of Medicine, Kaohsiung Medical University, Kaohsiung 807, Taiwan; 4 Research Center for Environmental Medicine, Kaohsiung Medical University, Kaohsiung 807, Taiwan

**Keywords:** hepatic steatosis, white blood cell, platelet

## Abstract

The incidence of hepatic steatosis is increasing globally, and it is important to identify those at risk to prevent comorbidities. Complete blood count is a simple, convenient, and inexpensive laboratory examination which can be used to obtain white blood cell (WBC) and platelet counts. The aims of this study were to investigate the relationships between WBC and platelet counts with hepatic steatosis, and whether WBC and platelet counts were associated with the severity of hepatic steatosis. We enrolled 1969 participants residing in southern Taiwan who took part in a health survey from June 2016 to September 2018 in this cross-sectional study. None of the participants were heavy alcohol users or had a history of hepatitis B or C. We collected laboratory data, and the severity of hepatic steatosis was determined by abdominal ultrasound. The overall prevalence rate of hepatic steatosis was 42.0%. There were significant trends of stepwise increases in WBC count (*p* < 0.001) corresponding to the severity of hepatic steatosis. After multivariable linear regression analysis, hepatic steatosis was significantly associated with high WBC count (coefficient β, 0.209; 95% confidence interval (CI), 0.055 to 0.364; *p* = 0.008) and high platelet count (coefficient β, 12.213; 95% CI, 6.092 to 18.334; *p* < 0.001); also, higher WBC counts corresponded with the severity of hepatic steatosis.

## 1. Introduction

The global prevalence of nonalcoholic fatty liver disease (NAFLD) is around 25% [1]; it has rapidly increased in Asia over the last decade from 25% to 34% [2]. NAFLD is a known risk factor for the development of hepatocellular carcinoma, advanced liver disease, and related complications, and it has also been strongly associated with cardiovascular diseases, metabolic syndrome, diabetes, and extrahepatic malignancy [3,4,5]. The pathogenesis of NAFLD is not fully understood, but insulin resistance and chronic inflammation have been shown to play vital roles [6]. Insulin resistance leads to abnormal triglyceride accumulation in hepatocytes, causing hepatic steatosis and then inflammation mechanisms including lipotoxicity and immune reaction, leading to nonalcoholic steatohepatitis (NASH) and activating hepatic stellate cells to cause hepatic fibrosis [7,8]. Accordingly, patients with NAFLD have been reported to have elevated levels of inflammatory biomarkers, including high-sensitivity C-reactive protein (CRP) [9], tumor necrosis factor-α (TNF-α) [10], and interleukin-6 (IL-6) [11]. Moreover, this chronic inflammation further worsens insulin resistance, leading to a vicious cycle of NAFLD progression [12]. Identifying NAFLD as early as possible is crucial to prevent the comorbidities of cardiovascular disease and metabolic syndrome [13,14]; therefore, it is important to predict the risk of NAFLD using simple and easily obtainable parameters.

White blood cell (WBC) count and platelet count are routinely measured in complete blood count. WBCs are cells of the immune system which play a role in differentiating diseases. An elevation in WBC count can be caused by bone marrow disorders or inflammatory diseases [15]. An increase in WBCs in the peripheral circulation from bone marrow is regulated by cytokines due to underlying inflammatory conditions, and an elevated WBC count has been associated with cardiovascular disease [16,17], metabolic syndrome [18,19], obesity [20], polycystic ovary syndrome [21], and insulin resistance [22]. Insulin resistance may cause hyperinsulinemia, leading to de novo hepatic lipogenesis and steatohepatitis [23]. The excess fatty acid then results in lipotoxic lipids and oxidative stress in hepatocytes, leading to liver necrosis [24].

Platelets participate in hemostasis by initiating a coagulation cascade at the site of interrupted endothelium; they also promote vascular inflammation [25] and tumor growth and survival [26]. An elevated platelet count can be reactive or be caused by hematologic malignancies or inherited syndrome (familial thrombocytosis). Reactive thrombocytosis can be caused by inflammatory diseases, iron deficiency, trauma, splenectomy or functional asplenia, acute blood loss, and neoplastic diseases [26]. An elevated platelet count has been associated with elevated CRP, IL-6, and erythrocyte sedimentation rate in cancer [27], and with IL-6 in inflammatory bowel disease and rheumatoid arthritis [28]. In addition, an elevated platelet count has also been associated with metabolic syndrome [29], cardiovascular disease [30], and insulin resistance [31]. Moreover, platelets will release platelet-derived growth factors, cytokines, and chemokines to activate the inflammation process to cause immune reactions, promote liver cirrhosis, and develop hepatocellular carcinoma [32,33,34,35]. In summary, the elevation of platelet counts is associated with CRP, IL-6, and ESR in various inflammatory diseases, and platelets play a role in the pathophysiology of liver disease.

Several studies have mentioned the association between WBC counts and NAFLD. They not only disclosed a positive correlation between WBC counts and the prevalence of NAFLD [36], but also established that elevation of WBC is a risk factor of NAFLD [22,37,38]. The association between platelet count and NAFLD has also been reported in previous studies [39,40,41,42,43,44], and the correlation is inconsistent. However, just few studies further differentiate the severity of hepatic steatosis and simultaneously analyze the counts of WBCs and platelets. In our study, we analyze WBC and platelet count at the same time and further elucidate that a stepwise increase in both corresponds to the severity of hepatic steatosis.

## 2. Materials and Methods

### 2.1. Participant Recruitment

This cross-sectional study was conducted in southern Taiwan from June 2016 to September 2018. The study protocol was approved by the Institutional Review Board of Kaohsiung Medical University Hospital (number: KMUHIRB-G(II)-20190011), and all participants gave informed consent before joining the study.

**Figure 1:**
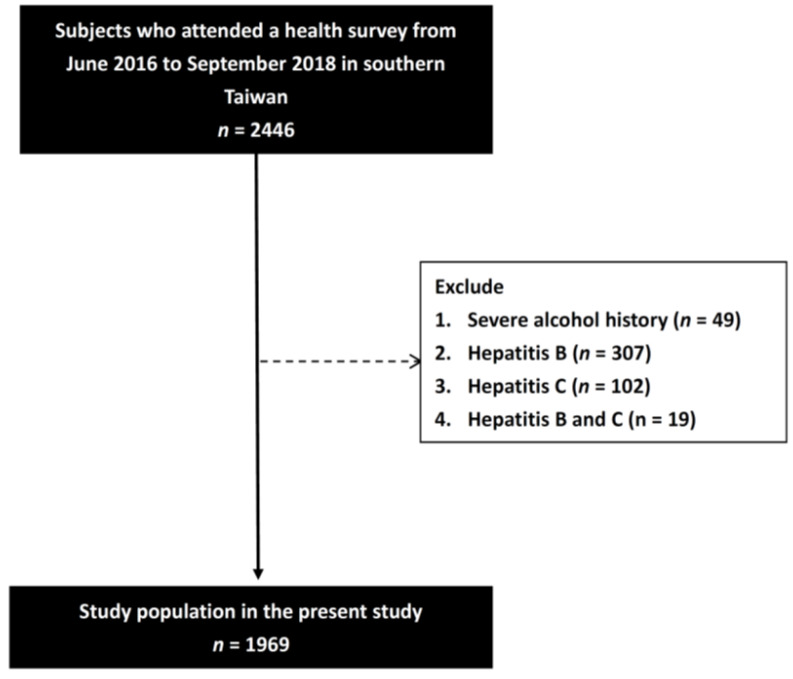
Flowchart of study population.

The participants were recruited from a health survey which was promoted through advertisements. They all underwent physical examinations, and an experienced physician recorded their clinical histories. Systolic blood pressure (SBP) and diastolic blood pressure (DBP) were also measured. All of the participants also underwent a face-to-face interview with a researcher, during which they completed a questionnaire asking about alcohol history. For men, heavy drinking is typically defined as consuming 15 drinks or more per week in his lifetime. For women, heavy drinking is typically defined as consuming 8 drinks or more per week in her lifetime. Participants who did not meet the above criteria and were not social drinkers were defined as having an alcohol history. Heavy alcohol users and those with a history of hepatitis B or C were excluded. In addition, hemochromatosis, Wilson disease, and autoimmune hepatic disorders were excluded. A total of 2446 participants with complete data were screened, of whom 477 were excluded due to a history of severe alcohol consumption (*n* = 49), hepatitis B (*n* = 307), hepatitis C (*n* = 102), and hepatitis B and C (*n* = 19). The remaining 1969 participants were analyzed (Figure 1).

### 2.2. Collection of Demographic, Medical, and Laboratory Data

Baseline data on lifestyle habits (smoking and alcohol consumption), medical history (diabetes mellitus (DM) and hypertension), and demographics (age and sex) were recorded. In addition, the following laboratory data were also recorded at baseline: WBC count, hemoglobin, platelet count, fasting glucose, triglycerides, total cholesterol, high-density lipoprotein (HDL) cholesterol, low-density lipoprotein (LDL) cholesterol, aspartate aminotransferase (AST), alanine aminotransferase (ALT), estimated glomerular filtration rate (eGFR), and uric acid. eGFR was calculated using the Chronic Kidney Disease Epidemiology Collaboration equation (CKD-EPI eGFR) [45].

### 2.3. Assessment of Hepatic Steatosis

Hepatic steatosis was defined as the presence of hepatic steatosis on liver ultrasonography that could not be explained by the secondary accumulation of hepatic fat or acute/chronic liver diseases, such as treatment with steatogenic drugs and severe alcohol use [46,47]. Liver ultrasonography was performed by experienced radiologists who were blinded to the participants’ biochemical data and clinical diagnoses. Hepatic steatosis was defined as the presence of at least two of the following: greater echogenicity in the liver compared to the kidneys, gradual attenuation of far-field ultrasound echo, vascular blurring, and diffuse increase in near-field ultrasound echo (‘bright liver’) [47,48]. Steatosis is graded as follows: absent (score 0), when the echotexture of the liver is normal; mild (score 1), when there is a slight and diffuse increase of liver echogenicity with normal visualization of the diaphragm and of the portal vein wall; moderate (score 2), in the case of moderate increase in liver echogenicity, with slightly impaired appearance of the portal vein wall and the diaphragm; severe (score 3), in the case of marked increase in liver echogenicity with poor or no visualization of portal vein wall, diaphragm, and posterior part of the right liver lobe [49].

### 2.4. Statistical Analysis

Continuous variables are presented as mean ± standard deviation or median (25–75th percentile), and categorical data are presented as number and percentage. Differences between categorical variables were compared using the chi-squared test, and differences between continuous variables were compared using the independent *t* test. Multiple comparisons among hepatic steatosis severity groups were performed using one-way analysis of variance followed by a Bonferroni-adjusted post hoc test. Spearman correlation was performed to identify the association between WBC and platelet counts with hepatic steatosis severity groups. Linear regression analysis was used to identify associations between WBC count and platelet count with hepatic steatosis. Significant variables in univariable analysis were entered into multivariable analysis. *p*-values < 0.05 were considered to be statistically significant. SPSS version 19.0 for Windows was used for all statistical analyses (SPSS Inc. Chicago, IL, USA).

## 3. Results

The mean age of the 1969 participants (764 males and 1205 females) was 54.9 ± 13.5 years, and the overall prevalence rate of hepatic steatosis was 42.0%. The clinical characteristics of the participants with and without hepatic steatosis are compared in Table 1. Compared to participants without hepatic steatosis, participants with hepatic steatosis were older, predominantly male, had higher prevalence of DM and hypertension, higher SBP, higher DBP, higher body weight, higher body mass index, higher WBC, higher hemoglobin, higher platelet, higher fasting glucose, higher triglyceride, lower HDL-cholesterol, higher LDL-cholesterol, higher AST, higher ALT, and higher uric acid.

### 3.1. Association between WBC and Platelet Counts with the Severity of Hepatic Steatosis

**Figure 2:**
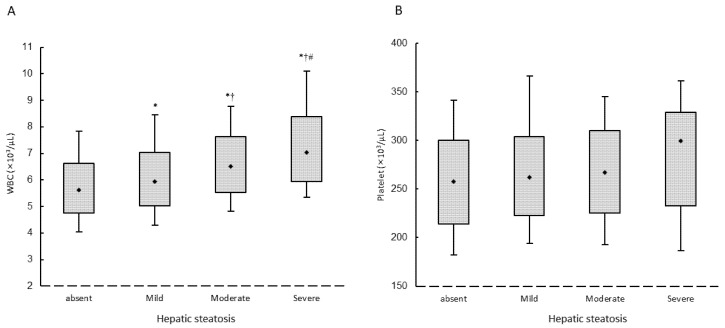
The relationship between the counts of white blood cell and the severity of hepatic steatosis (**A**), and the relationship between the counts of platelets and the severity of hepatic steatosis (**B**). Abbreviations: WBC, white blood cell. * *p* < 0.05 compared with non-hepatic steatosis; † *p* < 0.05 compared with mild hepatic steatosis; # *p* < 0.05 compared with moderate hepatic steatosis.

Figure 2 illustrates that the WBC and platelet counts among the four study groups were 5.6 (4.8–6.6), 5.9 (5.0–7.0), 6.5 (5.5,7.6), and 7.0 (5.9–8.4) × 10^3^/μL for WBC; and 258 (214–300), 262 (223–304), 267 (225–310), and 299.5 (233–329) × 10^3^/μL for platelets, respectively. The number of the participants of the four study groups were 1143, 437, 347, and 42, respectively. We observed the relationship between the counts of white blood cell and the severity of hepatic steatosis (A), and the relationship between the counts of platelets and the severity of hepatic steatosis (B). There was a significant trend for stepwise increases in the counts of WBC (ANOVA *p* < 0.001) corresponding to the severity of hepatic steatosis in study participants. There was also a statistical significant difference in individual groups in the WBC count.

### 3.2. Determinants of WBC Count Using Linear Regression Analysis

Table 2 shows the determinants of WBC count in the study participants. In the univariable linear regression analysis, hepatic steatosis, young age, male, smoking history, DM, high SBP, high DBP, high hemoglobin, high fasting glucose, high triglyceride, low HDL-cholesterol, high LDL-cholesterol, high AST, high ALT, high eGFR, and high uric acid were associated with high WBC count. As determined from multivariable linear regression analysis after adjusting for hepatic steatosis, age, gender, smoking history, DM, SBP, DBP, hemoglobin, fasting glucose, triglyceride, HDL-cholesterol, LDL-cholesterol, AST, ALT, eGFR and uric acid, hepatic steatosis (coefficient β, 0.209; 95% CI, 0.055 to 0.364; *p* = 0.008), young age (*p* < 0.001), female (*p* = 0.007), smoking history (*p* < 0.001), DM (*p* = 0.002), high hemoglobin (*p* = 0.001), high fasting glucose (*p* < 0.001), high triglyceride (*p* < 0.001), low HDL-cholesterol (*p* = 0.005), and high uric acid (per 1 mg/dL; coefficient β, 0.075; 95% CI, 0.020 to 0.129; *p* = 0.007) were significantly associated with high WBC count.

### 3.3. Determinants of Platelet Count Using Linear Regression Analysis

Table 3 shows the determinants of platelet count in the study participants. In the univariable linear regression analysis, hepatic steatosis, young age, female, non-DM, non-hypertension, low SBP, low hemoglobin, low fasting glucose, high total cholesterol, high HDL-cholesterol, high LDL-cholesterol, low AST, high eGFR, and low uric acid were associated with high platelet count. As determined from multivariable linear regression analysis after adjusting for hepatic steatosis, age, gender, DM, hypertension, SBP, hemoglobin, fasting glucose, total cholesterol, HDL-cholesterol, LDL-cholesterol, AST, eGFR and uric acid, hepatic steatosis (coefficient β, 12.213; 95% CI, 6.092 to 18.334; *p* < 0.001), young age (*p* < 0.001), female (*p* < 0.001), low hemoglobin (*p* < 0.001), high LDL-cholesterol (*p* = 0.049), high eGFR (*p* = 0.043), and high uric acid (per 1 mg/dL; coefficient β, 2.375; 95% CI, 0.154 to 4.596; *p* = 0.036) were significantly associated with high platelet count.

## 4. Discussion

NAFLD is a spectrum of liver disease, from simple hepatic steatosis to nonalcoholic steatohepatitis (NASH) with progressive hepatic inflammation and a higher risk of liver fibrosis and hepatic comorbidities. Cardiovascular events and extra-hepatic malignancies are the first and second most common causes of mortality in patients with NAFLD [13]. It is important to identify the presence and severity of NAFLD as early as possible to help prevent comorbidities. In this study, we observed that hepatic steatosis was associated with high WBC and platelet counts, and higher WBC counts corresponded with the severity of hepatic steatosis in 1969 participants residing in southern Taiwan.

The first important finding of this study is that the participants with hepatic steatosis had a higher WBC count. Furthermore, there was a significant trend of a stepwise increase in WBC count corresponding to the severity of hepatic steatosis. Lee et al. reported a positive correlation between WBC count and the prevalence of NAFLD, also showing an increased risk of NAFLD according to higher WBC count [36]. Yu et al. revealed that a higher WBC count is a risk factor of NAFLD, and that the stepwise increase in WBC count is a positive correlation with the risk of NAFLD combined with H pylori infection [38]. Further cohort studies also confirmed the association between an elevated WBC count and the risk of NAFLD [22,37]. In addition, Gokulakrishnan et al. reported an association between the severity of NAFLD and WBC count in south Indians in 2012 [50], which is consistent with our study result. Several mechanisms explain increased WBC count in hepatic steatosis [51]. First, insulin resistance causes hyperinsulinemia, leading to de novo hepatic lipogenesis and steatohepatitis [23]. The excess fatty acid then results in lipotoxic lipids and oxidative stress in hepatocytes, leading to liver necrosis. The apoptosis of hepatocytes produces damage-associated molecular patterns (DAMPs) and activates inflammatory signals, which then increases WBC count [24]. Second, interacting with the microbiome in the intestinal system and liver disease, so-called gut-liver axis has been established [3]. In health conditions, the permeability of the intestine is well controlled to avoid pathogen-associated molecular patterns—such as bacteria and bacterial toxic products including lipopolysaccharides—and endotoxin exposure via the hepatic portal system [52]. However, alcohol intake and a high-fat diet will cause dysbiosis [53], which leads to intestinal epithelial loosening and initiates intestinal inflammation by releasing inflammatory cytokines—such as interferon-gamma and interferon-alpha—through mucosal immune system cells [54]. The cellular toxic metabolites passing through the hyperpermeable intestinal barrier elicits liver inflammation and subsequently causes liver disease [54]. Third, as well as being an energy-storing organ, adipose tissue is the largest endocrine organ producing adiponectin, leptin, TNF-α, and IL-6, which leads to systemic inflammation and insulin resistance, thereby further exacerbating NAFLD [55]. In addition, a previous animal model showed that adipose death could trigger liver injury and inflammation [56]. Progressive inflammation plays an important role in the pathogenesis of the progression of NAFLD [57]. In addition, an increase in hs-CRP has been associated with the severity of NAFLD [58]. WBC count is also a surrogate marker of chronic inflammation, and it corresponded with the severity of hepatic steatosis in our study.

The second important finding of this study is the higher platelet count in the participants with hepatic steatosis. The association between platelet count and NAFLD is controversial. Several studies have observed a negative correlation between platelet count and the severity of NAFLD [39,40,41]. Possible mechanisms include liver cirrhosis with splenomegaly and decreased thrombopoietin production in patients with advanced liver disease [40]. However, Sung et al. reported a positive association between platelet count and the incidence of NAFLD [42]. In addition, Afagh et al. found that a higher platelet count was associated with more severe NAFLD [43], and the platelet count in patients with NASH has been reported to be higher than in patients without NASH [44]. Platelets play a role in liver regeneration after liver damage; however, they can also trigger the pathogenesis of liver fibrosis [59] by producing transforming growth factor-beta and platelet-derived growth factor, which activate fibrocompetent cells, notably hepatic stellate cells [57]. The fibrocompetent cells then remodel extracellular matrix components, leading to fibrosis [60]. In addition, platelets have been shown to induce innate and adaptive immune responses, thereby causing progressive liver damage in patients with chronic viral hepatitis and NAFLD [61]. Previous studies have also demonstrated an association between an elevated platelet count with metabolic syndrome [62] and insulin resistance [31]. Metabolic syndrome causes systemic chronic inflammation and an increase in IL-6, which are also associated with increased platelet production, and more risk factors for metabolic syndrome have been positively associated with a higher platelet count [62]. Insulin resistance has also been shown to increase platelet count through an increase in thrombopoietin production by excess adipose tissue [63]. The various mechanisms associated with platelets may have different effects on platelet count, and may be related to disease stage [59,60].

Another important finding of this study is that a high uric acid level was associated with high WBC and platelet counts. Hyperuricemia is defined as high levels of uric acid, an end-product of purine metabolism [64]. A diet rich in purine and alcohol consumption are major risk factors for hyperuricemia, and genetic factors are also important [64]. Uric acid produces free radicals, particularly fatty acids, which cause systemic inflammation and vascular injury [65], and subsequently cardiovascular disease, metabolic syndrome, chronic kidney disease, and insulin resistance [66]. Hyperinsulinemia has been shown to cause decreased uric acid secretion in renal tubules [67]. Two meta-analyses found that higher levels of uric acid were associated with the prevalence of NAFLD [68,69]. Moreover, a linear relationship between uric acid and the incidence of NAFLD has also been reported [70]. Furthermore, an observational study reported a higher uric acid level in non-obese patients with NAFLD compared with obese patients [71]. Hyperuricemia is accompanied by inflammatory markers, including IL-6, IL-18, hs-CRP, and TNF-α [72]. Su et al. reported a significant association between uric acid concentration and WBC count, but no significant association between uric acid and platelet count [73]. In addition, Liu et al. reported that hyperuricemia was associated with a higher WBC count, and that this association was independent of chronic kidney disease [74]. In contrast to these studies, Tayefi et al. reported positive correlations between platelet count and mean platelet volume with serum uric acid in newly diagnosed hypertensive patients [75].

There are several limitations to this study. First, we could not ascertain causal relationships or long-term clinical outcomes due to the cross-sectional design of this study. Prospective studies with NAFLD and immune function assessments and a longer follow-up period are needed to verify our results. Second, we did not evaluate sleeping or economic status in our analysis, both of which may also be associated with the development of NAFLD. Third, all of the participants in this study were Taiwanese, which may limit the generalizability of our results to other populations. Fourth, only participants who were willing to attend the study were included, making it considerably more difficult to interpret standard errors and confidence intervals. Fifth, insulin resistance is a hallmark of NAFLD and it plays a pivotal role in the pathogenesis of the disease. However, insulin level was not measured in this study. Therefore, we could not evaluate the association between insulin resistance and NAFLD. Sixth, we assessed the severity of NAFLD according to ultrasound findings. However, ultrasound of the liver is qualitative and may not be sufficient for scoring. In addition, future studies should include data on differentiated WBC count to further investigate impaired innate and acquired immune systems. Finally, the values of WBC and platelets are within the normal range in each group of participants. Therefore, it would be difficult to consider the measurement of WBC or platelet count helpful in the diagnosis of hepatic steatosis.

In conclusion, we found hepatic steatosis was associated with high WBC and plate-let counts, and higher WBC counts corresponded with the severity of hepatic steatosis; also, the pathogenesis may have been related to chronic inflammation. We suggest that WBC count and platelet count may play a role in stratifying hepatic steatosis.

## Figures and Tables

**Table 1:** Comparison of clinical characteristics among participants according to hepatic steatosis in study participants (*n* = 1969).

Characteristics	Hepatic Steatosis (−) (*n* = 1143)	Hepatic Steatosis (+) (*n* = 826)	*p*
Age (year)	54.3 ± 14.2	55.7 ± 12.6	0.027
Male gender (%)	35.9	42.9	0.002
Smoking history (%)	10.7	13.4	0.064
Alcohol history (%)	21.3	20.4	0.604
DM (%)	7.0	15.3	<0.001
Hypertension (%)	20.5	31.1	<0.001
SBP (mmHg)	128.8 ± 20.0	135.7 ±18.7	<0.001
DBP (mmHg)	75.7 ± 11.8	79.0 ± 10.9	<0.001
Body height (cm)	160.2 ± 8.4	160.8 ± 9.0	0.194
Body weight (kg)	60.7 ± 10.7	69.5 ± 12.7	<0.001
Body mass index (kg/m^2^)	23.6 ± 3.4	26.8 ± 3.7	<0.001
Laboratory parameters			
WBC (×10^3^/μL)	5.8 ± 1.5	6.5 ± 1.8	<0.001
Hemoglobin (g/dL)	13.8 ± 1.6	14.2 ± 1.7	<0.001
Platelet (×10^3^/μL)	261.5 ± 67.8	271.0 ± 68.5	0.002
Fasting glucose (mg/dL)	95.1 ± 21.0	106.8 ± 32.4	<0.001
Triglyceride (mg/dL)	87 (64–125)	134 (98–188.25)	<0.001
Total cholesterol (mg/dL)	199.4 ± 37.3	202.7 ± 37.7	0.059
HDL-cholesterol (mg/dL)	56.3 ± 13.9	48.2 ± 11.2	<0.001
LDL-cholesterol (mg/dL)	117.7 ± 32.8	123.6 ± 35.3	<0.001
AST (U/L)	25.2 ± 8.0	29.7 ± 14.2	<0.001
ALT (U/L)	20.4 ± 12.9	33.0 ± 23.5	<0.001
Creatinine (mg/dL)	0.94 ± 0.25	0.95 ± 0.28	0.381
eGFR (mL/min/1.73 m^2^)	89.7 ± 17.2	88.8 ± 15.3	0.206
Uric acid (mg/dL)	5.5 ± 1.5	6.0 ± 1.5	<0.001

Abbreviations: DM, diabetes mellitus; SBP, systolic blood pressure; DBP, diastolic blood pressure; WBC, white blood cell; HDL, high-density lipoprotein; LDL, low-density lipoprotein; AST, aspartate aminotransferase; ALT, alanine aminotransferase; eGFR, estimated glomerular filtration rate.

**Table 2:** Determinants of WBC count using linear regression analysis.

Variables	Univariable		Multivariable	
	Unstandardized Coefficient β (95% CI)	*p*	Unstandardized Coefficient β (95% CI)	*p*
Hepatic steatosis	0.667 (0.522, 0.812)	<0.001	0.209 (0.055, 0.364)	0.008
Age (per 1 year)	−0.013 (−0.019, −0.008)	<0.001	−0.027 (−0.037, −0.018)	<0.001
Male (vs. female)	0.469 (0.320, 0.617)	<0.001	−0.270 (−0.467, −0.073)	0.007
Smoking history	1.353 (1.134, 1.573)	<0.001	1.004 (0.778, 1.230)	<0.001
Alcohol history	0.174 (−0.007, 0.354)	0.059	-	-
DM	0.682 (0.445, 0.919)	<0.001	0.434 (0.160, 0.708)	0.002
Hypertension	0.117 (−0.052, 0.286)	0.175	-	-
SBP (per 1 mmHg)	0.006 (0.002, 0.009)	0.002	0.004 (−0.001, 0.009)	0.088
DBP (per 1 mmHg)	0.014 (0.007, 0.020)	<0.001	−0.001 (−0.009, 0.007)	0.827
Laboratory parameters				
Hemoglobin (per 1 g/dL)	0.208 (0.163, 0.252)	<0.001	0.087 (0.033, 0.140)	0.001
Fasting glucose (per 1 mg/dL)	0.010 (0.008, 0.013)	<0.001	0.006 (0.003, 0.009)	<0.001
Triglyceride (log per 1 mg/dL)	1.904 (1.610, 2.197)	<0.001	0.775 (0.412, 1.138)	<0.001
Total cholesterol (per 1 mg/dL)	0 (−0.002, 0.002)	0.847	-	-
HDL-cholesterol (per 1 mg/dL)	−0.027 (−0.032, −0.021)	<0.001	−0.009 (−0.015, −0.003)	0.005
LDL-cholesterol (per 1 mg/dL)	0.003 (0.001, 0.005)	0.003	0.002 (0, 0.004)	0.086
AST (per 1 U/L)	0.013 (0.006, 0.019)	<0.001	−0.005 (−0.016, 0.006)	0.343
ALT (per 1 U/L)	0.016 (0.012, 0.019)	<0.001	0.005 (−0.001, 0.012)	0.122
eGFR (per 1 mL/min/1.73 m^2^)	0.006 (0.002, 0.010)	0.008	−0.005 (−0.013, 0.003)	0.203
Uric acid (per 1 mg/dL)	0.193 (0.146, 0.240)	<0.001	0.075 (0.020, 0.129)	0.007

Values expressed as unstandardized coefficient β and 95% confidence interval (CI). Abbreviations: DM, diabetes mellitus; SBP, systolic blood pressure; DBP, diastolic blood pressure; WBC, white blood cell; HDL, high-density lipoprotein; LDL, low-density lipoprotein; AST, aspartate aminotransferase; ALT, alanine aminotransferase; eGFR, estimated glomerular filtration rate.

**Table 3:** Determinants of platelet count using linear regression analysis.

Variables	Univariable		Multivariable	
	Unstandardized Coefficient β (95% CI)	*p*	Unstandardized Coefficient β (95% CI)	*p*
Hepatic steatosis	9.482 (3.382, 15.583)	0.002	12.213 (6.092, 18.334)	<0.001
Age (per 1 year)	−1.580 (−1.792, −1.368)	<0.001	−1.448 (−1.836, −1.060)	<0.001
Male (vs. female)	−31.035 (−37.073, −24.997)	<0.001	−20.475 (−28.290, −12.659)	<0.001
Smoking history	0.415 (−8.980, 9.809)	0.931	-	-
Alcohol history	−2.313 (−9.768, 5.142)	0.543	-	-
DM	−20.151 (−29.970, −10.333)	<0.001	−3.306 (−14.601, 7.990)	0.566
Hypertension	−19.206 (−26.129, −12.283)	<0.001	1.473 (−5.783, 8.729)	0.691
SBP (per 1 mmHg)	−0.231 (−0.383, −0.079)	0.003	0.154 (−0.003, 0.310)	0.055
DBP (per 1 mmHg)	0.258 (−0.003, 0.519)	0.053	-	-
Laboratory parameters				
Hemoglobin (per 1 g/dL)	−8.608 (−10.426, −6.790)	<0.001	−8.251 (−10.410, −6.093)	<0.001
Fasting glucose (per 1 mg/dL)	−0.197 (−0.309, −0.086)	0.001	−0.003 (−0.132, 0.126)	0.968
Triglyceride (log per 1 mg/dL)	11.627 (−0.954, 24.209)	0.070	-	-
Total cholesterol (per 1 mg/dL)	0.097 (0.017, 0.177)	0.018	0.009 (−0.152, 0.171)	0.911
HDL-cholesterol (per 1 mg/dL)	0.284 (0.061, 0.507)	0.013	−0.179 (−0.449, 0.091)	0.193
LDL-cholesterol (per 1 mg/dL)	0.130 (0.041, 0.218)	0.004	0.167 (0.001, 0.334)	0.049
AST (per 1 U/L)	−0.488 (−0.755, −0.222)	<0.001	−0.062 (−0.319,0.195)	0.635
ALT (per 1 U/L)	−0.046 (−0.204, 0.112)	0.568	-	-
eGFR (per 1 mL/min/1.73 m^2^)	1.132 (0.956, 1.309)	<0.001	0.333 (0.010, 0.656)	0.043
Uric acid (per 1 mg/dL)	−4.479 (−6.436, −2.522)	<0.001	2.375 (0.154, 4.596)	0.036

Values expressed as unstandardized coefficient β and 95% confidence interval (CI). Abbreviations: DM, diabetes mellitus; SBP, systolic blood pressure; DBP, diastolic blood pressure; WBC, white blood cell; HDL, high-density lipoprotein; LDL, low-density lipoprotein; AST, aspartate aminotransferase; ALT, alanine aminotransferase; eGFR, estimated glomerular filtration rate.

## Data Availability

Data may be available upon request from interested researchers. Please send data requests to Szu-Chia Chen, PhD, MD, Division of Nephrology, Department of Internal Medicine, Kaohsiung Medical University Hospital, Kaohsiung Medical University.
